# Best practices for engaging with affected communities: chronic hepatitis B as a case study

**DOI:** 10.1186/s40249-025-01288-7

**Published:** 2025-03-04

**Authors:** Thomas Tu, Nafisa Yussf, Lien Tran, Kim Ngo, Su Wang, Adi Mondel, Isabelle Purcell, Jacki Chen, Wendy Lo, Bright Ansah, Kenneth Kabagambe, Soumen Basu, Dee Lee, Supa Chantschool, Chris Munoz, Ivana Dragojevic, Marko Korenjak, Fiona Borondy-Jenkins, Yasmin Ibrahim, Beatrice Zovich, Chari Cohen

**Affiliations:** 1 Storr Liver Centre, The Westmead Institute for Medical Research, The University of Sydney at Westmead Hospital, Westmead, NSW Australia; 2 Centre for Infectious Diseases and Microbiology, Sydney Infectious Diseases Institute, The University of Sydney at Westmead Hospital, Westmead, NSW Australia; 3 HepBCommunity.Org, Sydney, NSW Australia; 4 Hepatitis B Voices Australia, Melbourne, VIC Australia; 5 Hepatitis B Foundation, Doylestown, PA USA; 6 World Hepatitis Alliance, London, UK; 7 Cooperman Barnabas Medical Center, Florham Park, NJ USA; 8 ASHM Health, Sydney, Australia; 9 Rutgers Health, Robert Wood Johnson Medical School, Piscataway, NJ USA; 10 Taiwan Hepatitis Information & Care Association, Princeton, NJ USA; 11 The National Organisation for People Living With Hepatitis B, Kampala, Uganda; 12 Hepatitis Patient Forum, Liver Foundation, West Bengal, India; 13 Inno Community Development Organisation, Guangdong, China; 14 Hep B Companion, London, UK; 15 Yellow Warriors Society of the Philippines, Quezon City, Philippines; 16 European Liver Patients’ Association, Brussels, Belgium

**Keywords:** Lived experience, Consumer engagement, Expert consensus, Patient advocacy, Community research, Policy development, Hepatitis B, Collaborative partnership, Patient representation

## Abstract

Hepatitis B is the single most common cause of liver cancer, affecting > 250 million people worldwide (mostly in resource limited communities) and killing > 1 million people annually. The condition is marked by poor rates of diagnosis (14%) and treatment (8% of eligible individuals). As with many health conditions, engagement with the affected community is crucial for designing, promoting, and advocating for effective solutions in the health system. However, engagement with the affected community remains difficult in many instances due to variable understanding of the roles, capacities, and expertise of people with lived experience. Through community-led consensus, we provide here several practical approaches for how public health, clinical, scientific, industrial, and policy-making bodies should engage with the hepatitis B affected community. These expert consensus practices have been developed by people living with hepatitis B and/or advocating for them. We suggest that these practices should be incorporated into any engagements with communities affected by hepatitis B and can be generalisable to other health conditions.

**Graphical Abstract:**

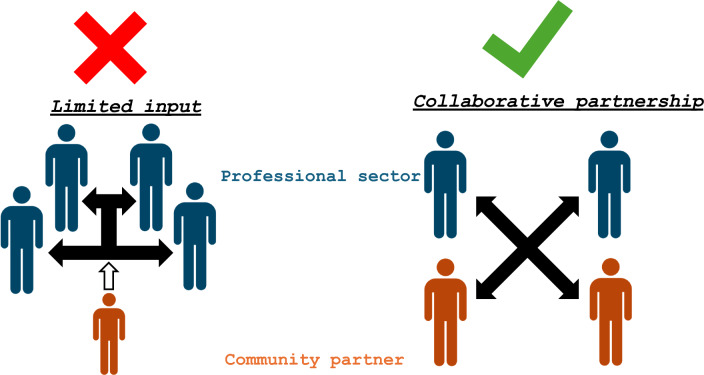

## Background

Chronic hepatitis B affects 254 million people worldwide and causes 1.1 million deaths every year through liver cancer or cirrhosis [1], which is equivalent to more than one person dying every minute. Even with the safe and effective vaccination for prevention, hepatitis B will continue to affect our societies until at least 2080 [2]. Once a chronic infection is established in a person, it is likely to persist throughout their entire life. If left unmanaged, people living with hepatitis B have a significantly increased lifetime risk of dying from liver-related disease. Importantly, they also experience other psychosocial impacts [3, 4], including: fear and anxiety surrounding disease progression or transmitting the infection to others; financial instability from health costs; stigma and discrimination; and rejection by society [5].

Chronic hepatitis B has shamefully low rates of diagnosis with 14% of people worldwide with hepatitis B aware of their status [6]. These figures have not significantly improved in the last decade, stymied by multiple factors:

- Poor access to trustworthy education and limited awareness of hepatitis B and its health impacts;
- Stigma (from other community members and health care providers, and internal self-stigma);
- Fear of discrimination (e.g., by employers, colleagues, or immigration departments [5]);
- Negative prior experiences with the health care system;
- Limited opportunities for affordable/accessible diagnostics;
- Lack of access to appropriate hepatitis B testing, treatment and care;
- Difficulties in navigating the health care system or understanding test results;
- Confusion around treatment guidelines for both patients and clinicians; and
- Lack of political will and funding for resources to address hepatitis B as a public health issue commensurate to its disease prevalence.

The complexity in the experience of hepatitis B has been a major bottleneck in developing efficient strategies for elimination efforts and improvements in community health. Developing effective approaches is highly challenging with hepatitis B: the strong societal stigma and discrimination associated with the condition prevents people from engaging with the health care system, which is not always accessible to the often-underserved communities affected by hepatitis B. Moreover, there is lack of wider understanding of the intersectional perspectives surrounding hepatitis B as physical and psychosocial issues to engage the diverse populations it affects.

Ongoing and meaningful community engagement, co-design, co-production, and decision-making in a sustainable partnership is required to improve health outcomes on a broader scale in a systematic, effective, and continuous manner. The hepatitis B affected community has only recently been engaged with professional scientific and clinical bodies with any significant frequency. Increasingly, the importance of community involvement in achieving elimination goals is becoming better understood across the field. Indeed, significant improvements in public response have been seen in several other chronic health conditions with strong affected community voices, such as mental health, HIV, and hepatitis C.

A key shortcoming is the limited understanding within the professional sector in how to effectively engage with the affected community. To address this, we the authors (as people with lived experience of hepatitis B and representatives of patient advocacy groups) have developed 13 expert consensus practices to enable respectful and effective partnership with communities affected by hepatitis B (Table 1). This collection of practices is aimed to help to build bridges between affected communities and the sector to improve health and wellbeing outcomes. This aritcle represents a consensus viewpoint of members from community-led organisations: Hepatitis B Voices Australia (Australia); HepBCommunity.org (Australia); ELPA (Europe-wide); Yellow Warriors Society Philippines Inc. (The Philippines); Hep B Companion (United Kingdom); THICA (USA); Community Advisory Board of the Hep B PAST project (Australia); World Hepatitis Alliance (international members) and the Community Advisory Board of the Hepatitis B Foundation (international members).

**Table 1:** Thirteen expert consensus practices for partnering with the HBV-affected community

#	Principles
1	Collaborate with community partners early and throughout the project design, providing opportunities for actionable input into the process
2	Remunerate community partners appropriately for their expertise
3	Invite more than one community partner
4	Recruit community partners through established community groups
5	Provide contract agreements that are easy to understand and equitable
6	Choose the right level of engagement
7	Ensure accessibility of briefing information ahead of meeting
8	Recognise the technical support needed to optimally engage
9	Be mindful of community partners during a meeting, actively seek out their counsel, and engage with them
10	Ensure safety and respect boundaries regarding confidentiality, privacy, and disclosure
11	Use trauma-informed practices and practice cultural safety
12	Provide emotional support to community partners
13	Debrief with community partners and include them in follow-up steps

We intend this collection of expert consensus practices to inform the professional sector (including researchers, clinicians, decision makers, public health workers, governments, academics, non-profit institutions, and industry partners; herein referred to as investigators) about best practices that should be followed when considering engagement with people affected by hepatitis B (herein referred to as community partners). We believe that adherence to these practices will facilitate respectful, sustainable, and effective partnerships to develop practical solutions to the huge impacts of chronic hepatitis B.

## Best practices for engaging with the affected community

### Collaborate with community partners early and throughout the project design, providing opportunities for actionable input into the process

Engaging the affected community should not be a rubber-stamping process, but an opportunity to design approaches and materials that are meaningful, relevant, productive, and effective. Community input into the conception and design phases (when the approaches are still flexible) can inform appropriate and effective initiatives, spending resources into pursuing programs that are better tailored and fit for purpose. This early involvement can facilitate:

- The validation or correction of assumptions about the lived experience of hepatitis B;
- Using accessible, appropriate, and respectful language, particularly when referring to people living with hepatitis B;
- The identification of health system concerns and suggestions to improve/fix those concerns from a wider range of perspectives; and
- Mitigation of costly blind spots (e.g., factors impacting implementation in culturally diverse communities);
- The reduction of the risks of ineffective (or even counter-productive) approaches.

Early engagement could also improve the quality of feedback, both in the short- and long-term. Early and extended engagement could promote trust and improve buy-in from the community partners, who may become more invested in the success of a program that they have spent more time shaping compared to a one-time review. Moreover, if the feedback is seen as actually influencing the direction of a program (rather than a simple approval or a sign-off at the end of the process), then community partners are more likely to spend more effort in considering new ideas and impacts of a program. Finally, such respectful engagement acknowledges the time and effort of community partners and less likely to lead to feelings of tokenism, resentment and belittlement.

There can be a bias that the community partner is not seen as equal in expertise compared to others, and their input may be considered as of lesser importance. Efforts should be made to ensure that there is equivalency, respect for all expertise, and true partnership. For example, in a guideline group or other committee, community partners should have equal consideration (voting, authorship, etc.). In a training or speaking event, community partners should be listed as an expert and considered as a member of the speakers’ faculty.

### Remunerate community partners appropriately for their expertise

Planning for this partnership should include budgeting for consultation into funding structures and grant proposals so that time and resources of community partners and organisations can be covered.

Regardless of formal qualifications, people with lived experience bring expertise. Indeed, no one is in a better position to provide informed, first-person perspective and insights into the impacts of hepatitis B than the people who have decades of experience in living with the condition. The participation and engagement with affected community should therefore be remunerated appropriately. Several tools have been developed to standardise the level of compensation, considering people’s experience and expertise (e.g., fair market calculators [7] or renumeration guidelines [8]).

It is common to invite people in the profession (e.g., clinicians and researchers) to participate in panel discussions, committees, and lectures with little to no remuneration. Arguably, this could be considered as community service associated with their paid position within the public sector. However, this practice should not extend to the community partners, who often volunteer their time and efforts to improve care for common good. The affected community often takes time off their paid work and may have few financial mechanisms to support their advisory work.

As a standard, expertise from the affected community should be paid as consultants and include the extra time that may be required for any additional briefing or training needed to bring the community partner up to speed. Non-monetary remunerations (gift cards, conference registration, or travel) may be negotiated in the case there is no budget for any advisory time. It is only through this approach that lived experience expertise can be embedded as a sustainable service for the sector.

When appropriate, engagements should be used as an opportunity for professional development, e.g. upskilling the community worker to further improve ongoing engagement: this ‘cultural bridge’ between research/intervention and community groups should be fostered.

### Invite more than one community partner

Communities affected by hepatitis B are large and heterogeneous, affecting people of different backgrounds, genders, ethnicities, cultures, experiences, geographical location, political views, and expertise. Effective programs aiming to reach all people with hepatitis B should therefore incorporate diverse perspectives. A simple way to achieve this is to ensure multiple members of the affected community are involved throughout the design and implementation processes, and to ensure that these members represent the broad spectrum of people with hepatitis B by considering the diversity related to culture, gender identity, age, socioeconomic status, and hepatitis B experiences.

Not only would this improve the quality and relevance of feedback for the investigator, but this incorporation of multiple community partners would also have positive benefits for the community partners themselves before, during, and after meetings. It can be intimidating, unsafe and overwhelming for people outside of the professional hepatitis B sector to provide feedback to those with high levels of specialist expertise. Speaking out can be difficult when assumptions are made by others in the meeting about a group’s understanding of complex jargon and terminology (that is difficult for non-experts to follow) during the discussion. The presence of other people at the same level of expertise significantly improves confidence to speak up, support each other and seek additional support required. This, in turn, promotes simplification of ideas and equity, ensuring that all community partners can fully engage in the discussion.

Moreover, if the discussion involves traumatic or emotionally stressful topics (such as the impacts of the diagnosis of a terminal disease, struggles with migration, or the torment felt from discrimination and stigma), peer support allows community members to vent, support one another, and debrief about the emotional impacts. This is particularly true for a highly stigmatized disease like hepatitis B, where only a few of those aware of their status feel comfortable enough to share their experiences publicly. It is important that program coordinators and meeting facilitators/chair recognise this, ask the community partners what support they need before, during and after engagement and provide these supporting systems effectively.

### Recruit community partners through established community groups

A complementary approach to ensure that broad experiences are considered is recruiting people with lived experience through established community groups. Community groups often share experiences, providing community partners with a broader perspective of impacts affecting people with hepatitis B outside of their own personal experience. This strengthens their capacity for advocacy and fuller representation of the expansive breadth of experiences associated with hepatitis B.

Moreover, the quality of feedback and advice is likely to be higher when engaging through community groups (compared to recruiting isolated individuals) as it is standard practice within these groups to provide representatives with background education in hepatitis B biology, clinical pathways, and current progress, as well as upskilling in storytelling, public speaking, and framing of feedback.

While overheads associated with community groups may increase costs in recruitment, community groups are generally not-for-profit and feed this funding back into programs that help build their public speaking capacity and education of those living with the disease, and other community support. Building good-will within the community extends the sustainability of these advisory services.

Community groups also provide a support framework for the community partners. This includes negotiation for remuneration, governance structures, legal review of contracts, selection of appropriate candidates for a specific advisory need, and emotional support during and after the engagement. This addresses the perception of potentially asymmetric power in the dealing and ensures that respectful and equal partnerships are developed between parties. Community groups also have access to educational materials specifically designed and tailored for members of the non-specialist community and could assist in briefing the community partner beforehand. In summary, working together with community groups is likely to improve effectiveness and efficiency in the co-design processes.

### Provide contract agreements that are easy to understand and equitable

People with lived experience frequently contribute as private individuals and not an organizational entity with its own legal department. To remedy this asymmetrical relationship, investigators should help the individual to become properly informed and to understand the agreement before signing. The terms and conditions should be presented as a proposal that is reasonable and equitable for any individual citizen or consumer (not an organization). If the language in the contract agreements is complex (e.g. legal jargon), investigators should describe the proposed agreement in a way that a layperson can understand, as well as offer to explain, answer questions, and make amendments when appropriate. Engagement through community groups can facilitate these conversations on a more equal basis.

### Choose the right level of engagement

The level of engagement should be carefully considered and will depend on a given program’s objectives, goals and resources. The International Association for Public Participation has a public participation spectrum that is an international standard for engagement and describes engagement along a continuum [9]. At the lower end, there is simply *informing* people of work that is being undertaken. At the other end, there is *co-design and co-production*, where people with lived experience have every opportunity to inform the work and its outcomes. We recommend that co-production approach to ensure initiatives are co-led by people living with and affected by hepatitis B.

As mentioned earlier, meetings and panel discussions can be intimidating for people who are not professionals within a sector. This is particularly exacerbated when a meeting requires understanding of highly technical concepts or is not carried out in the first language of the community partner (which is often the case for the highly diverse communities affected by hepatitis B).

Engagement with less urgency and fewer on-the-spot requirements may be easier and more expedient to participate in for the affected community. Beyond large group meetings, investigators should consider engagement in other formats (e.g., a document review in one’s own time, a survey, an interview with questions provided beforehand, a one-on-one meeting, or a prerecorded speeches). By providing adequate time for preparation, the quality of feedback is likely to be improved.

### Ensure accessibility of briefing information ahead of meeting

As people with lived experience of hepatitis B are generally not professionally trained in the sector (indeed they should not be for the investigator to gain a representative perspective of the affected population), briefing the community partner to be on the same page as the rest of the investigator’s working group may require additional considerations. Investing in this initial groundwork to prepare the community partner reduces the time during a meeting to explain basic concepts, gives them the opportunities to be better involved in the discussion, and ultimately improve the quality of feedback and overall contributions. Several aspects should be considered when providing this briefing information, including:

#### Linguistic aspects

Many people from the diverse affected communities do not speak English as a first language. Even if most communication across the engagement is in English, materials provided in the non-technical language or community partners’ first language(s) are useful in conveying information in an accessible way. This includes avoiding the use of acronyms that may be commonplace in the clinical/scientific sector but not widely known by the wider public. Moreover, particular focus should be made to avoid stigmatising language when discussing hepatitis B, given the widespread experience of stigma and discrimination due to this condition.

#### Conceptual aspects

Considering community partners may not be formally trained in the sector, information should be provided at a level that is conceptually accessible. This includes limiting the use of jargon and matching the provided information to the frameworks of health. For example, in some cultures, there is no word for “hepatitis” and “inflammation” and as such there needs to be an appropriate reframing or translation of the concepts in these instances. Moreover, graphs, statistics, and other standard approaches to presenting scientific data may be difficult for the hepatitis B community to interpret quickly. Explanation of these data should be appropriately presented to be accessible to the community.

#### Administrative aspects

To a certain extent, the use of technical language is unavoidable given the nature of medical research and implementation. In these circumstances, it is helpful to provide a longer lead time between the provision of reading documents and when feedback is expected. Opportunities to ask questions, peer support, and receive briefings should also be provided.

To this point, investigators should also be mindful when imposing short deadlines that community partners are typically advising outside of their own professional and personal lives. For example, expecting a fast turnaround for review of complicated documents is not conducive to meaningful outcomes, more likely to yield lower quality feedback, and will likely frustrate community partners and impact the professional relationship.

Information should be provided not only about the specific topic at hand, but also the structure and roles within the group. Titles within industry and academia are often unfamiliar to people outside these structures. Moreover, discussing the specific areas and types of feedback needed during the briefing will help the community partner to prepare for and stay attentive during the consultation. Ensuring clarity in the meeting’s objectives, outcomes and processes can significantly improve feedback.

### Recognise the technical support needed to optimally engage

Other practical aspects are important to consider in the lead up to and during an advisory meeting. As community partners are generally acting on an ad hoc basis rather than as part of an institution, many software platforms that are taken for granted may not be available (e.g., paid subscriptions to video-conferencing, word-processing, and journal access platforms).

Moreover, community members may not be fluent or comfortable in using sector standard software and may require support prior to the event. Internet access may be of poor quality for people in low resource settings, which can limit video/audio connections or even connecting to the meeting. Where possible, several different approaches to provide feedback should be available and supported. Moreover, ensuring a stable internet connection during the meeting enables more meaningful contributions from the community partner.

During a meeting, particular considerations should be made regarding sound quality and visibility of each speaker. This helps with understanding what the speaker is saying, especially for attendees with hearing issues or who are not native English speakers. All participants should be reminded at the beginning of a meeting to speak slowly and clearly, and to avoid using abbreviations and technical terms with which community members may not be well versed.

### Be mindful of community partners during a meeting, actively seek out their input, and engage with them

Event hosts and meeting participants have a responsibility to make everyone (especially the community partner) feel included in the meeting. This includes ensuring that community partners are provided with opportunities to contribute and voice their feedback. In unfamiliar formats and structures, community partners may not feel comfortable and be hesitant to provide input.

Approaches in how to appropriately provide these opportunities must be carefully considered and discussed with the community partner prior the meeting. For example, an explicit agenda item on consumer input may be welcomed by some people, but to others may inadvertently imply that community partners are excluded from the rest of the discussion. Alternatively, explicitly calling out community partners during the discussion could either be well received or interpreted as singling them out. The most respectful approach would be for the chair to discuss exactly how the community partners wish to engage in the discussion prior to the meeting and be empathetic to their needs. This collaboration could best identify where and how the community partner can also offer input for the rest of the agenda.

Community partners may comment on unexpected topics. Often, investigators have a set perspective on the topics of input from community partners (e.g., whether programs are acceptable to patients or communities). However, the lived experience perspective is important beyond that. Investigators should be open to the perspective of lived experience bringing in new research questions, interpreting studies and research, assessing current approaches, designing surveys or programs or even offering ideas for new policies. Specific platforms or dedicated time in meetings could be allocated for discussion of these topics.

### Ensure safety and respect boundaries regarding confidentiality, privacy, and disclosure

Given the association of chronic hepatitis B and stigma throughout the community, shared information by the community partner must be handled with appropriate care. Disclosure of the condition can lead to real impacts including self-stigma, embarrassment, loss of employment, imperilled relationships, etc. Community partners may be sharing traumatic and intimately personal experiences during the engagement and should have total control over the extent of detail and distribution of that story. Therefore, the scope of disclosure should be discussed prior to engagement and their choices should be respected.

Privacy, level of disclosure, recording, retention of information, and post-event distribution of what is shared during engagement must be discussed (and agreed upon) prior and confirmed after the engagement. In the case of a live event whether virtual or in-person, provide questions ahead of time and respect boundaries on what not to talk about, if it is outside the person’s comfort level. Investigators should always provide community partners with the option to limit identifying information, recordings of the engagement, and any aspects of the engagement that are distributed publicly and privately (e.g., part of promotional materials). Investigators should be aware that identifying information may be inadvertently shared (e.g., last names exposed when using a personal video-chatting software) and appropriate risk controls should be planned. Finally, community partners should be given the opportunity to review the media release consent forms and investigators should offer option to amend it for the specific scenario.

### Use trauma-informed practices and practice cultural safety

Hepatitis B predominantly affects underserved, culturally and linguistically diverse communities, as well as indigenous communities. It is important to recognise that many of the affected communities come from colonised countries and may have experienced war, displacement trauma, stigma, discrimination and racism [10]. Additionally, community partners may also be living within closed immigrant communities and vulnerable to migration-related issues. Therefore, people working with affected communities should complete cultural safety and trauma-informed training before engagements (including First Nations cultural safety and those for specific multicultural communities).

It is important to understand the differences between cultural awareness, cultural sensitivity and cultural safety. **Cultural awareness** is recognising and understanding the cultural differences that exist between people. **Cultural sensitivity** goes a step further by showing respect and appreciation for these differences and being mindful not to offend or harm others. **Cultural safety** is ensuring the environment feels safe for all individuals by actively addressing power imbalances and creating inclusive practices that honour cultural identities and experiences. Cultural safety focuses on recognizing and addressing power imbalances, individual and institutional biases, and systemic inequities that affect marginalised people. It requires ongoing reflection, self-awareness, and a commitment to equitable and inclusive practices by organisations and individuals.

Investigators should also ensure that affected communities are reflected in the investigator’s workforce across all levels to build community trust and provide culturally appropriate care. Undertaking cultural safety training and hepatitis B specific training helps health professionals to understand that certain topics of discussion can be inadvertently insensitive to needs of affected community or could trigger memories of trauma (e.g., emotional impacts surrounding diagnosis, migration experience, discrimination, disclosure of status, etc.).

### Provide emotional support to community partners

Sharing lived experiences can be uncomfortable, triggering, and daunting, requiring appropriate emotional and social support. Discussions prior to engagement should be conducted to determine the support that community partners may require, ensuring these supports are in place in a timely manner. This may include pre-brief, debriefs and/or referrals to external support (e.g. community organisations, psychologists, etc.). Consistent with the above, this support should be culturally appropriate for the community partner.

Particularly for virtual events, where in-person interactions may not be immediately available after the meeting, emotional support should be provided. When such virtual events end, the individual is left alone to process the various dynamics and emotions that may have arisen. While not every participant may experience this level of emotional work, providing support throughout as an option can be helpful. Emotional support can be provided through peers or community groups, who should be appropriately referred to and acknowledged.

While negative emotions and impacts may be unavoidable during the engagement due to the nature of bringing light to traumatic experiences, limiting and addressing these will ensure ongoing commitments and buy-in from the community for partnership projects.

### Debrief with community partners and include them in follow-up steps

Debriefing after the engagement serves several important purposes: bringing a sense of closure to the event; helping the community partner to decompress from sharing what may be a stressful and vulnerable experience; giving both parties the opportunity to exchange feedback and perspectives on the engagement; and allowing the community partner to understand their impact on the program or the field, in general. Debriefing also allows any concerns or negative experiences to be raised so that community engagement can be continually improved for all parties. Debriefing helps participants redigest the information acquired and potentially provide more advice once the experience is internalised and processed. Offering timely, respectful feedback with acknowledgement of community partner’s contribution can provide positive support and encouragement.

Where possible, a summary of the engagement (including discussion points and key actions) should be provided for record-keeping and agreement on the accuracy of what was discussed. Finally, community partners should be provided with the outcome of the consultation to see how their advice was used and ensure that their intentions are accurately captured in the final context. Community partners should be offered the opportunity to be included as participants, authors or reviewers in any meeting reports, publications or dissemination efforts.

## Conclusions

Strong partnership with the affected community is crucial for promoting and advocating for improved and more effective action from all stakeholders in the clinical, scientific, political, and public health responses not only for hepatitis B, but many other conditions.

Along with other professional experts, people with lived experience bring expertise and should be engaged for the unique first-person perspectives they bring to discussions and program designs. Indeed, community engagement has been highlighted by the WHO as necessary to “advance equitable progress towards universal health coverage, while promoting transparency and accountability.”[11]. Empowering communities and civil society is also one of WHO’s five strategic directions in the guiding framework to implement hepatitis elimination globally. [12]

We believe that adopting these best practices will facilitate equitable, respectful, and sustainable relationships between professional sectors and the associated affected communities, leading to programs that are meaningful and effective in improving the lives of people and communities affected by hepatitis B and other health conditions.

## Data Availability

Not applicable.

## Abbreviations

WHO: World Health Organization
HIV: Human immunodeficiency virus
ELPA: European liver patients’ association
THICA: Taiwan hepatitis information & care association
Hep B PAST: Partnership approach to sustainably eliminating chronic hepatitis B in the northern territory
